# Endoscopic antralplasty for severe gastric stasis after wide endoscopic submucosal dissection in the antrum

**DOI:** 10.1007/s12328-016-0640-0

**Published:** 2016-03-22

**Authors:** Yoshiko Ohara, Takashi Toyonaga, Akiko Tanabe, Hiroshi Takihara, Shinichi Baba, Taro Inoue, Wataru Ono, Fumiaki Kawara, Shinwa Tanaka, Takeshi Azuma

**Affiliations:** Division of Gastroenterology, Department of Internal Medicine, Graduate School of Medicine, Kobe University, Kobe, Japan; Department of Endoscopy, Kobe University Hospital, 7-5-1 Kusunoki-cho, Chuo-ku, Kobe, Japan; Department of Endoscopy, Kishiwada Tokushukai Hospital, Kishiwada, Japan; Department of Gastroenterology, Ikoma City Hospital, Ikoma, Japan

**Keywords:** Endoscopic antralplasty, Endoscopic submucosal dissection, Gastric stasis

## Abstract

A 75-year-old female underwent esophagogastroduodenoscopy, revealing a widely spreading tumor occupying the anterior wall, lesser curvature, and posterior wall of the antrum and lower body. Endoscopic submucosal dissection was performed and resulted in more than five-sixths circumferential antral mucosal resection. One month later, she complained of nausea, vomiting, and abdominal distention. Endoscopy showed residual food in the stomach and deformation of the antrum with traction toward the contracted scar in the lesser curvature. The pyloric ring could not be seen from the antrum although the endoscope was able to pass easily beyond the area of deformation and the pyloric ring was intact. Despite repeated endoscopic balloon dilations, the patient’s symptoms remained refractory. The problem was speculated to be not due to any potential stricture but to antrum deformation resulting from the traction force toward the healing ulcer. We hypothesized that an additional countertraction force opposite the previous ESD site might resolve the problem, and ESD of approximately 2.5 cm size was performed in the greater curvature of the antrum. Along with development of a scar, traction toward the greater curvature was added, and the pyloric ring could be observed on repeat esophagogastroduodenoscopy. The symptoms were also gradually ameliorated. Afterwards, the endoscopic findings have now been unchanged during 7 years of follow-up.

## Introduction

Endoscopic submucosal dissection (ESD) techniques have recently been improving and have been increasingly applied for the treatment of non-neoplastic diseases, such as perioral endoscopic myotomy (POEM) for achalasia [1], anti-reflux mucosectomy (ARMS) for reflux esophagitis [2], and gastric perioral endoscopic myotomy (G-POEM) for pyloric stenosis and gastroparesis [3, 4]. These new treatment modalities have gradually replaced the surgical treatments that used to be done for these diseases.

We experienced a case in which severe gastric stasis was caused after wide ESD in the antrum and lower body of the stomach because of antral deformation by a contracted scar. The patient was successfully treated with additional ESD on the contralateral side of the  original ESD scar. We present this case as the first report of a new and promising method to treat gastric stasis after antral ESD.

## Case report

**Fig. 1:**
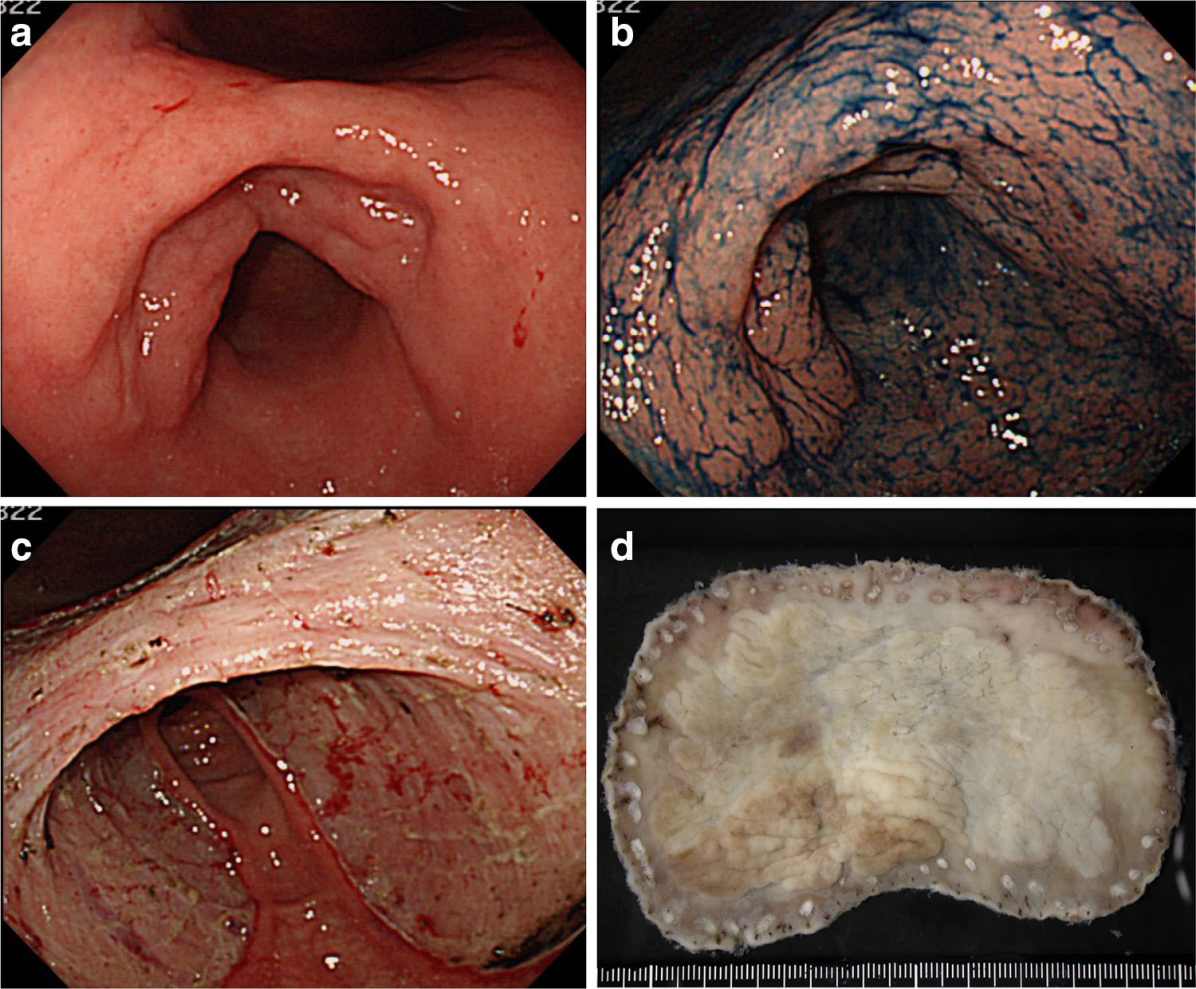
**a** Widely spreading tumor in the antrum and lower body. **b** Indigo carmine staining of the tumor. **c** Artificial ulcer after ESD with a mucosal defect of more than five-sixths circumference in the antrum and lower body. **d** Resected specimen measuring 110 × 70 mm

**Fig. 2:**
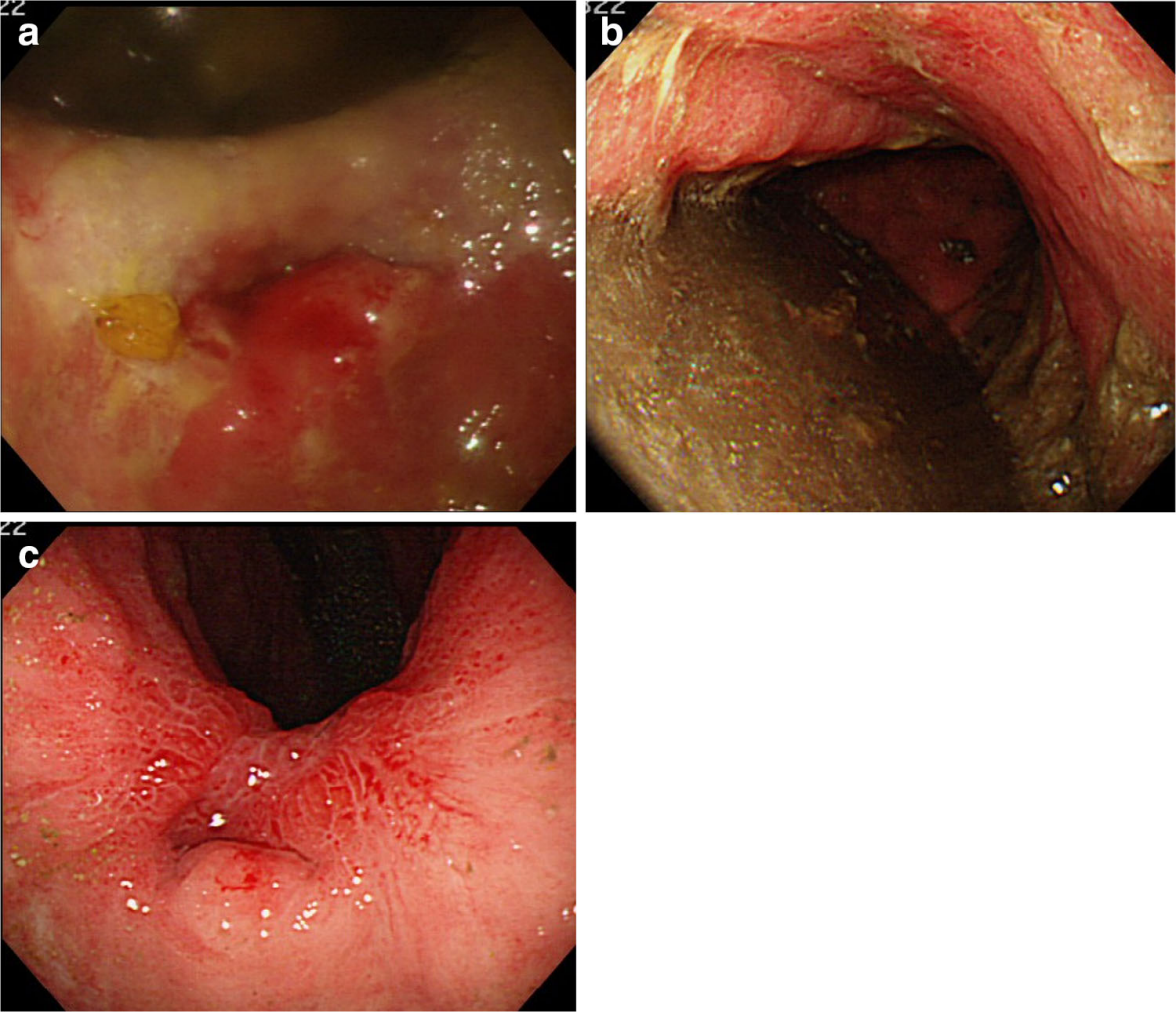
**a** Ulcer 1 month after ESD. The antrum is deformed, and the pyloric ring cannot be seen from the antrum. **b** Residual food in the stomach because of deformation of the antrum. **c** Ulcer scar at 4 months after ESD. The pyloric ring still cannot be visually confirmed from the antrum because of the deformation

**Fig. 3:**
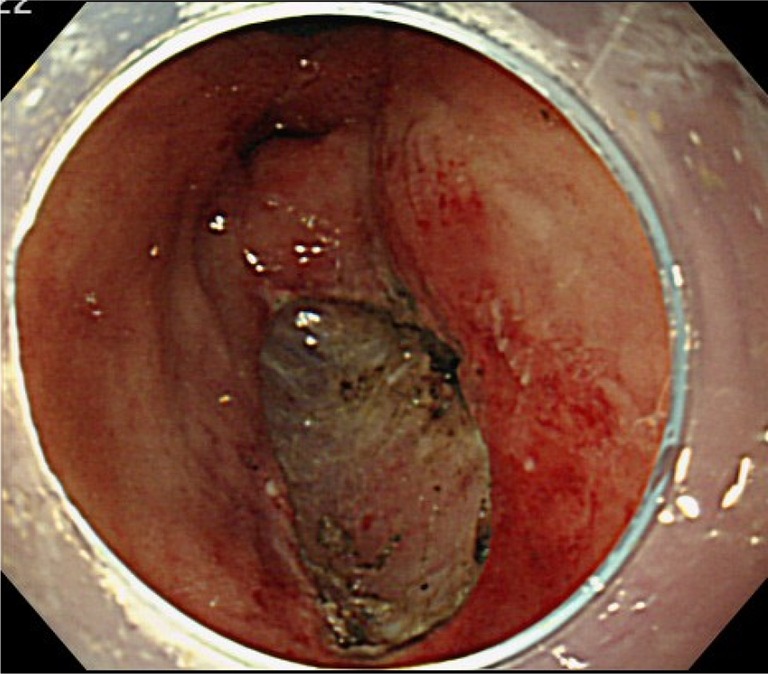
ESD was performed on the greater curvature in the antrum 4 months after the first ESD

**Fig. 4:**
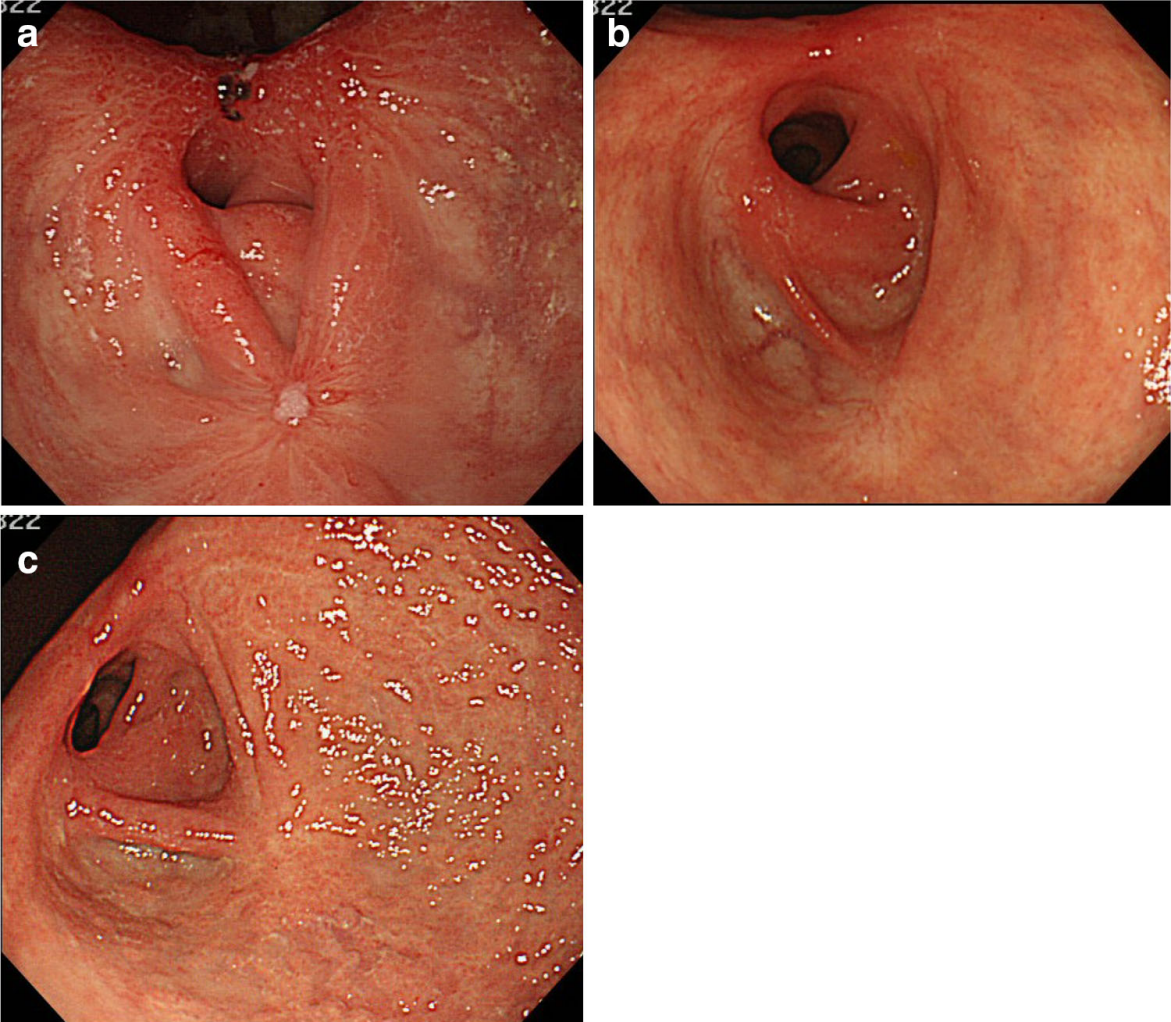
**a** Endoscopic view 1 month after the endoscopic antralplasty. The ulcer added traction toward the great curvature, and the pyloric ring became observable from the antrum. **b** Endoscopic view 1½ years after the antralplasty. **c** Endoscopic view 7 years after the antralplasty

A 75-year-old female consulted a nearby clinic because of abdominal pain. She underwent esophagogastroduodenoscopy, which revealed a widely spreading tumor occupying the anterior wall, lesser curvature, and posterior wall of the antrum and lower body (Fig. 1a, b). The biopsy showed a large, well-differentiated adenocarcinoma with no sign of deep submucosal invasion. ESD was indicated, and the patient underwent ESD using the Flush knife-BT (DK2618JB; Fujifilm Medical Co., Ltd., Tokyo, Japan) [5], without any intraoperative adverse events. The procedure took 79 min. The mucosal defect in the antrum and the lower body spanned more than five-sixths of the antral circumference (Fig. 1c). Histopathological observation showed a well-differentiated adenocarcinoma, 88 mm in size, invading 200 μm deep into the submucosa (Fig. 1d). The resected margin was tumor-negative. Although the resection was non-curative according to the JGES criteria [6], the patient did not choose to undergo additional surgery and was closely observed. One month later, the patient complained of symptoms of gastric stasis, including nausea, vomiting, abdominal distention, and loss of appetite. Endoscopy showed deformation of the antrum with traction toward the contracted scar in the lesser curvature and residual food in the stomach (Fig. 2a–c). The pyloric ring could not be seen from the antrum; however, the endoscope was able to pass easily beyond the area of deformation, which suggested that there was no severe physical stricture. The pyloric ring was also confirmed to be intact. Despite repeated endoscopic balloon dilations (EBDs), the patient’s symptoms did not improve. Two months later, she suffered from a full thickness tear in the upper stomach after vomiting, which was cured conservatively by gastric decompression and fasting. However, gastroparesis continued. These episodes suggested that the problem was not due to physical antrum stricture but due to deformation of the antrum resulting from the traction force toward the lesser curvature generated by the healing ESD induced ulcer. We hypothesized that an additional counter-traction force might resolve the problem. With the aim of releasing the deformation, ESD of approximately 2.5 cm in size was performed in the great curvature of the antrum (Fig. 3). Along with development of a scar, traction toward the great curvature was added, and the pyloric ring could be observed from the antrum 1 month later (Fig. 4a). The symptoms also gradually ameliorated, and finally resolved. During subsequent 7 years of follow up, the endoscopic findings have not changed (Fig. 4b, c), and no tumor recurrence or gastric stasis symptom has been observed.

## Discussion

Stricture can develop after ESD for large esophageal and prepylorus lesions, and EBD is widely recognized as an effective strategy for its treatment [7, 8]. On the other hand, in the antrum, deformation after ESD can lead to gastric stasis even when the stricture is mild or not present, as was observed in the present case. Our case demonstrates that the antral deformation can be treated by additional ESD on the contralateral side from the original ESD, with maintenance of this endoscopic and clinical effect for a significant amount of time.

A previous report suggested that contralateral mucosal incision and steroid injection were useful for treatment of stenosis after wide ESD in the antrum [9]. The authors suggested that the strategy’s mechanism depended on induction of granulation by the incision and steroid injection aiding in the widening of the antrum, which differed from the mechanism of the present method. The authors also mentioned that a combination with oral steroid administration resulted in a better prognosis. However, steroid treatments may sometimes be problematic; specifically steroid injections to the ulcer may raise the risk of late perforation [9], and oral steroid administration after ESD may cause severe infectious disease [10].

The present method utilizes conventional ESD techniques with no use of steroids. Creation of a counter-traction force against the original contracted ESD scar may help resolve the deformity and gastric stasis secondary to wide ESD.

It must be noted that the limitation of the present method is that it has only been performed in one case. It remains to be confirmed whether the present method can be applied to other patients, including those who undergo wide ESD in the greater curvature of the antrum, or whether there are potential complications such as a risk of developing stricture.

However, endoscopic antralplasty may be an alternative therapeutic option for post-ESD gastric stasis and worth considering prior to surgery because of its reduced invasiveness and ability to preserve the stomach compared with surgery. This method may help patients suffering from gastric stasis after wide ESD and avoid unneeded surgery.
